# Superiority of a Novel Multifunctional Amorphous Hydrogel Containing *Olea europaea* Leaf Extract (EHO-85) for the Treatment of Skin Ulcers: A Randomized, Active-Controlled Clinical Trial

**DOI:** 10.3390/jcm11051260

**Published:** 2022-02-25

**Authors:** José Verdú-Soriano, Marisol de Cristino-Espinar, Silvia Luna-Morales, Caridad Dios-Guerra, Javier Caballero-Villarraso, Paloma Moreno-Moreno, Antonio Casado-Díaz, Miriam Berenguer-Pérez, Ipek Guler-Caamaño, Olga Laosa-Zafra, Leocadio Rodríguez-Mañas, José Luis Lázaro-Martínez

**Affiliations:** 1 Department of Community Nursing, Preventive Medicine, Public Health and History of Science, Faculty of Health Sciences, University of Alicante, 03690 Alicante, Spain; miriam.berenguer@ua.es; 2 Nursing Department, Reina Sofia University Hospital, 14004 Córdoba, Spain; ms.cristino.sspa@juntadeandalucia.es; 3 Maimonides Institute of Biomedical Research of Cordoba (IMIBIC), Reina Sofía University Hospital, University of Córdoba, 14004 Córdoba, Spain; silvialu67@gmail.com (S.L.-M.); cdiosguerra@gmail.com (C.D.-G.); bc2cavij@uco.es (J.C.-V.); paloma.moreno.moreno.sspa@juntadeandalucia.es (P.M.-M.); ipek.guler@kuleuven.be (I.G.-C.); 4 Occidente Health Center, Córdoba and Guadalquivir Health Management Area, 14005 Córdoba, Spain; 5 Department of Nursing, Faculty of Medicine and Nursing, University of Cordoba, 14004 Córdoba, Spain; 6 Department of Biochemistry and Molecular Biology, Faculty of Medicine and Nursing, University of Cordoba, 14004 Córdoba, Spain; 7 Clinical Management Unit of Endocrinology and Nutrition, Reina Sofia University Hospital, 14004 Córdoba, Spain; 8 Consortium for Biomedical Research in Frailty & Healthy Ageing, (CIBERFES), Institute of Health Carlos III, 28029 Madrid, Spain; olga.laosa@salud.madrid.org (O.L.-Z.); leocadio.rodriguez@salud.madrid.org (L.R.-M.); 9 Geriatric Research Group, Biomedical Research Foundation at Getafe University Hospital, 28905 Getafe, Spain; 10 Department of Geriatrics, University Hospital of Getafe, 28905 Getafe, Spain; 11 Diabetic Foot Unit, University Clinic of Podiatry, Complutense University of Madrid, 28040 Madrid, Spain; diabetes@ucm.es

**Keywords:** diabetic foot, EHO-85, pressure ulcer, randomized active-controlled trial, venous leg ulcer

## Abstract

This 8-week, multicenter, randomized, active-controlled, observer-blinded clinical trial was designed to demonstrate the accelerating effect on wound healing of the novel *Olea europaea* leaf extract hydrogel (EHO-85) by comparing it to a widely used amorphous hydrogel. Results showed that EHO-85 significantly accelerated wound healing, regardless of ulcer etiology (pressure, venous leg or diabetic foot) and prognosis, doubling the median wound area reduction compared with a reference amorphous hydrogel (79.4% vs. 39.7%; difference: −39.7%, 95% CI: −71.1 to −21.3%; *p* < 0.001). The intention-to-treat analysis was conducted on 195 patients from 23 Spanish health centers/nursing homes. This novel treatment balances the ulcer microenvironment by modulating reactive oxygen species and pH. These actions complement the moistening and barrier functions inherent to amorphous hydrogels, whilst also conferring EHO-85 its documented granulation formation and pain relief properties. Furthermore, efficacy was achieved safely and in a cost-efficient manner due to its multi-dose format, which reduced the amount of product needed by 85.8% over 8 weeks compared to single-use hydrogel. The present randomized controlled trial is a relevant milestone in evidence-based practice for being the first to demonstrate (i) the effectiveness of an amorphous hydrogel in accelerating wound healing and (ii) the superiority of a specific hydrogel over another.

## 1. Introduction

Chronic wounds are of increasing frequency globally because of ageing populations and the rising prevalence of obesity, diabetes, and vascular disease [1]. The most common types are lower limb ulcers or diabetic etiology and skin lesions related to dependence, such as pressure ulcers. It has been estimated that in a population of 1 million people, 3500 people would have a chronic wound or skin ulcer, of which 525 would have a lesion with an evolution >12 months [2]. Those injuries represent an important burden for patients and society in terms of quality of life, resource consumption, and direct and indirect costs [2,3]. Moreover, patients with chronic wounds not only have increased morbidity [4], but also their mortality risk is higher than that of the general population, independent of age, sex, and ulcer etiology [3].

Treatment of chronic wounds must be holistic, acting on the etiology and providing appropriate local therapy. Multiple agents are available for topical treatment, such as hydrocolloids, polyurethane elastomers, polymeric foams, silicones, and hydrogels [5]. However, despite its potential for wound healing promotion [6,7], little attention has been paid to modulation of the ulcer microenvironment, although wound-bed preparation is considered an essential step towards healing [8].

Accordingly, several meta-analyses and systematic reviews have documented that the use of different topical agents or dressings does not promote or accelerate healing in pressure ulcers (PU) [9,10], venous leg ulcers (VLU) [11], or diabetic foot ulcers (DFU) [12]. More importantly, for currently available hydrogels, systematic reviews in the Cochrane Library do not provide evidence of superiority of any specific product over any other products [13].

Due to the increasing challenge of the problem posed by skin ulcers, new dressings with better clinical results are required. Wounds in the usual practice are currently treated by focusing on their signs and symptoms, with little attention directed to other underlying phenomena occurring in the ulcer microenvironment, such as tissue hypoxia, inadequate moisture, persistent inflammation, defects in the extracellular matrix, raised levels of alkalinization and ROS or the presence of metalloproteases. In this context, multifunctional dressings able to modulate wound microenvironment throughout the entire healing process besides acting as physical protective barriers against infection are currently of great interest. 

In line with the above, supported by in vitro and in vivo studies showing its properties and mechanisms of action [14], EHO-85, a novel multifunctional amorphous hydrogel containing *Olea europaea* leaf extract, was developed. This new product has the moisturizing and barrier-function properties inherent to amorphous hydrogels. However, in addition, it has been designed to decrease pH and to downregulate reactive oxygen species (ROS) within the ulcer microenvironment, thereby preparing the wound bed to speed-up the healing process.

The inclusion of the *Olea europaea* leaf extract deals with the need for a non-enzymatic ROS scavenger able to avoid the excessive and sustained increase in oxidative stress on the wound bed over time, which slows down or lead to a chronification of the healing process. Polyphenols such as those contained in the *Olea europaea* leaf extract, especially oleuropein, act protecting and promoting the viability of the cell types involved in the structure and regeneration of the skin, such as dermal fibroblasts and keratinocytes [14].

EHO-85 ability to promote wound healing was further supported by in vivo studies conducted in a murine model of impaired wound healing (BKS. Cg-m +/+ Leprdb). EHO-85 treatment showed superior wound healing rates compared to standard amorphous hydrogels [14]. This would confirm the relevance of the holistic approach to wound management made possible by the novel EHO-85 gel.

Therefore, with this background, the objective of the present randomized controlled trial was to test the healing-promoting effect of the novel EHO-85 gel by comparing it to a widely used amorphous hydrogel.

## 2. Patients and Methods

### 2.1. Design

The study had a prospective, multicenter, parallel-group, randomized, researcher-blind design, with the aim of evaluating the non-inferiority and potential superiority of EHO-85 compared to a product that has been used widely for many years. The study was approved by the Ethics Committee of Córdoba (Córdoba, Spain) and by the Spanish Agency of Medicines and Medical Devices Products (AEMPS) (PS/CR 623/17/EC). The trial was undertaken in accordance with the ISO 14155 Standard “Clinical investigation of medical devices for human subjects—Good clinical practice” and the Declaration of Helsinki. All participants provided written informed consent. 

### 2.2. Patients (Inclusion and Exclusion Criteria)

Patients of both sexes were eligible if they were at least 18 years old and were diagnosed with VLU; category II (partial thickness) or III (full thickness with skin loss) PU according to the European Pressure Ulcer Advisory Panel (EPUAP) [15] or grade I or II DFU according to the Wagner scale [16] of neuropathic origin. All ulcers had to have an evolution between 1 and 36 months, and an area between 1 cm^2^ and 199 cm^2^. If more than one ulcer was present, the ulcer that best met the selection criteria was selected (target ulcer).

Overall, exclusion criteria were HbA1c >9.5%, serum albumin <2.5 g/dL, severe renal failure, liver failure, connective tissue disease, systemic infection, local wound infection, and pregnancy and breast feeding. Patients were also excluded if they were receiving systemic corticosteroids, immunosuppressants, tumor necrosis factor (TNF) inhibitors for rheumatoid arthritis therapy, or PPAR-gamma agonists for diabetes treatment. In parallel, VLU and DFU had to have posterior tibial and/or pedal pulse present and the Ankle Brachial Pressure Index (ABPI) had to be at least 0.8. Furthermore, other specific exclusion criteria for each type of ulcer were defined (see Table S1). In case wound debridement and/or infection control were needed, therapy onset was to be postponed from 24 h to 72 h after debridement completion.

### 2.3. Investigational Product, Comparator, and Treatment Description

The EHO-85 gel is a class IIb medical device in the form of an amorphous hydrogel. Among other constituents, it is composed of: *Olea europaea* leaf extract, due to its free radical regulation ability [14]; biosaccharide-gum 1 (an anionic polysaccharide) and glycerin, as hydrating and self-emulsifying agents; a carbomer (crosslinked acrylic acid polymer) with excellent rheological properties which contributes to provide an isolating and protective barrier for the ulcer bed; gluconolactone and sodium benzoate, included to avoid microbiological contamination; and EDTA, chosen for its antimicrobial and antibiofilm properties. The combination of these components, together with the capability of the formulation to reduce the alkalinity of the ulcer bed through its slightly acidic pH (range 5.0–5.5), gives the EHO-85 gel the ability to modulate the wound environment, thus promoting and speeding up the healing process [14].

Regarding the organoleptic characteristics of the EHO-85 gel, its appearance was that of a homogeneous and translucent gel, odorless and with a pale-yellow color. Among its physical and chemical characteristics are (i) its slightly acid pH (pH range 5.0–5.5), (ii) its viscosity (spd 5v5 rpm, 22 °C) of 35,000–50,000 centipoise, and (iii) its density (20 °C) of 1.05–1.10 g/mL. According to the European Standard BS EN 13726-1, in relation to the absorbency properties of EHO-85, it possesses a significant moistening capacity (type D according to Method 3.4.) and is partially dispersible (Method 3.7.).

A thin layer, from 2 to 3 mm, was to be applied on the wound. Up to 5 mm was to be applied in the case of cavitated ulcers. Despite being a sterile product, a single tube was to be reutilized in several applications on the same wound since its formulation incorporates a preservative.

The positive control product was another amorphous hydrogel (VariHesive^®^, ConvaTec, Barcelona, Spain), a product of reference that is commercialized in other countries under other brand names such as Duoderm Hidrogel Activo^®^, Duoderm Hydroactive Sterile Gel^®^, Duoderm Gel^®^, or GranuGel^®^. It is composed of carboxymethylcellulose sodium salt, pectin, propylene glycol, and water. It is a single-use product applied following the manufacturer’s instructions. 

Treatment duration was set at 8 weeks unless complete healing was achieved previously. Product applications were to be carried out 3 days a week (24 applications in total at most) and, whenever possible, on alternate days. 

With the exception of the treatment product being applied, the care protocol was identical in both groups. The target wound had to be cleaned using only sterile saline. Subsequently, a silicon foam dressing was to be applied (Mepilex^®^, Molnlycke, Gothenburg, Sweden). Any other general or local treatments were not allowed.

In cases where, due to the exudate level or the clinical aspect of the wound, dressing changes needed to be >3 per week, the same treatment protocol would apply to the complementary wound care visits, except for the application of the study or comparator hydrogel. The investigator was to indicate this, together with the reason and the number of wound cures finally performed.

Compression therapy was mandatory among patients with VLU. Elastic compression bandaging (Indacrep^®^, Inda, Barcelona, Spain) was to be applied over the secondary dressing. The bandage was changed every 24 or 48 h depending on the needs of each patient. Similarly, patients with PU had to comply with a standardized repositioning regime. Patients with DFU used felted foam in combination with appropriate footwear. Prior to the start of the study, researchers explained in detail to patients and caregivers the protocol to be followed.

In the case of infection in the ulcer during the study, the frequency of cleanses and secondary dressing changes were to be intensified. Additionally, nanocrystalline silver dressing (Acticoat^®^/Argencoat^®^; Smith&Nephew, Barcelona, Spain) was to be applied over the ulcer until remission of the infection. The Clinical Investigation Plan did not provide for treatment products to be discontinued. However, the final decision was at the discretion of the investigator who could discontinue treatment for this reason upon justification.

### 2.4. Randomization and Stratification

To increase the internal validity of the trial, the study was designed to be randomized, stratified and assessor-blinded in order to minimize selection and confounding biases, with the aim that all extraneous factors would be equally distributed in the two study groups and that the only relevant difference between the groups being compared might be the treatment. Stratification considered ulcer etiology on a first level (PU, VLU or DFU) and subsequently ulcer duration (6 months cutoff) [17,18,19] and ulcer area (10 cm^2^ cutoff) [18,19]. Stratified randomization was performed using Research Electronic Data Capture Software REDCap^®^ version 7.06 (Research Electronic Data Capture System, Vanderbilt University, Nashville, TN, USA) (REDCap^®^), programmed for this purpose by the Innovation Department of the Instituto Maimónides de Investigación Biomédica de Córdoba (IMIBIC, Córdoba, Spain; IMIBIC Innovation Department).

### 2.5. Procedures

Any patient presenting with a PU, VLU, or DFU who met the selection criteria could be approached for inclusion in the study, irrespective of previous local treatment. After obtaining the patients or legal representatives’ written informed consent to participate in the trial, demographic parameters, patient’s medication and medical, surgical, and ulcer history were documented at the screening visit. The treatment of the studied ulcer during the prior month, etiology, duration, measurement, and location, among other items, were recorded. Subsequently, patients eligible for the study were enrolled and allocated randomly to either the test (EHO-85) or the control (VariHesive^®^, Convatec, Barcelona, Spain) amorphous hydrogel. At the screening visit, researchers confirmed that all the eligibility criteria were met. Ulcers were evaluated by the research nurse every 2 weeks until week 8. However, the last visit could be performed prematurely if there was complete healing, withdrawal, or discontinuation. Ulcer evolution was assessed through digital pictures taken at the therapy onset (Visit 1) and at every biweekly visit. In the case of complete wound closure, a picture documenting complete epithelialization was to be taken on that precise date. At each visit, 2 pictures of the wound of at least 8 megapixels were to be taken after cleansing the wound with saline and immediately sent to be reviewed, independently of the sponsor, by an experienced and software-trained main investigator who had not participated in the trial by applying treatments and who was blinded to the dressing type. Pictures of the wounds were to show a standardized label including date, patient code, and a millimeter rule used to enable digital planimetry wound and granulation tissue area measurements (Pictzar Pro^®^ version 7.5.1; Advanced Planimetric Services LLC, Elmwood Park, NJ, USA) [20].

Ulcer pain or discomfort was self-assessed at Visit 1 and at the last available visit using the “pain/discomfort” scale of the EuroQol Group EQ-5D-5L questionnaire. With this scale, patients rated their pain or discomfort from 1 (no pain or discomfort) to 5 (extreme pain or discomfort) [21]. At each biweekly visit, wound evaluations, which included other parameters such as exudate level, signs of infection, or adherence to the clinical investigation plan, were repeated. Between the biweekly investigator’s assessments, every product application and its completion date were recorded by the research nurse, as well as any application discontinuation and its cause. Investigators were also required to notify, at any time, all changes in medication, deviations from the clinical investigation plan or unexpected adverse events (whether related to treatment or not). 

### 2.6. Efficacy Endpoints 

The primary study outcome was relative Wound Area Reduction (WAR) calculated as [(Area_last_–Area_t0_)/Area_t0_] × 100 and expressed as a percentage (%). Area_last_ was the last available measure obtained. Subgroup evaluations were performed to assess if there were differences in the magnitude of the effect of the treatments on WAR according to ulcer type: VLU, PU, and DFU.

Secondary endpoints, designed to confirm whether the EHO-85 gel promotes and/or accelerates the wound healing process, included: absolute WAR, calculated as [(Area_last_ – Area_t0_)/Area_t0_] and expressed in sq mm; wound healing rate [(Area_last_ – Area_t0_)/(t_last_ – t_0_)], expressed in sq mm per day of treatment; rate of complete ulcer healing and the mean time to reach full epithelization; percentage of patients with a relative WAR ≥ 40% at last available measure and the mean time to reach the WAR ≥ 40% goal. WAR ≥ 60% and WAR ≥ 80% were also assessed during the post hoc analysis.

Other secondary endpoints included ulcer pain or discomfort relief, granulation tissue formation, and the amount of product needed to fulfill the 8 weeks of treatment calculated as the number of 15 g packs used per patient during the study. 

### 2.7. Safety Endpoints

Adverse events (AEs), as well as discontinuations and/or withdrawals due to AEs, were recorded at all evaluation visits, including nature, severity, time of onset, duration, degree of relationship to the study treatment, and a description of any action and/or pharmacological treatment undertaken to handle the event.

### 2.8. Data Collection and Database

An electronic data collection form based in REDCap^®^ was used, using the Data Process Center of the University of Córdoba, Spain. Data were anonymized and measures were taken to assure the confidentiality of patients’ data.

### 2.9. Statistical Analysis

Statistical analyses were conducted by an independent third party (IMIBIC Innovation Department) using R 4.0.3 software (R Foundation for Statistical Computing, Vienna, Austria). All analyses were conducted on an “intent-to-treat” (ITT) population, defined as all randomized patients who received the allocated treatment at least once. In addition, a sensitivity analysis was performed to detect significant variations between the safety, ITT, and “per protocol” test samples. There were no relevant differences or imbalances between treatment groups for any of the parameters evaluated.

#### 2.9.1. Sample Size Calculation

Sample size was calculated to document the non-inferiority of the EHO-85 gel compared to control treatment on the 8-week WAR from baseline value. Based on previous preclinical experience in murine models with the novel gel, the expected reduction in the experimental group was hypothesized to be slightly superior to comparator, mainly due to its ability to modulate the wound microenvironment. The expected superiority of the EHO-85 gel on WAR was fixed at 5%, with a 35% standard deviation, as observed in similar trials found in the literature for existing treatments [6,22]. Accordingly, a pre-specified non-inferiority margin equal to 10% was set, as it is considered to be the minimum clinically relevant difference [22]. Assuming normality and considering a one-sided α significance level equal to 0.025, 174 subjects were necessary to achieve a power of 80% (87 subjects for each of the control and intervention groups). In anticipation of possible withdrawals and dropouts, estimated at 15%, it was concluded that a sample of at least 200 patients would be necessary, 100 in each group. All ulcer types (VLU, PU and DFU) should have a minimum representation of 10 patients.

In case non-inferiority could be documented, in order to demonstrate superiority of the EHO-85 gel, given the previously estimated sample, the Clinical Investigation Plan considered that assuming an α risk fixed at 0.05, it would be necessary to obtain a difference on the WAR of both groups of at least 15% at the end of the study to achieve a power of 80%, assuming normality. This difference was in line with that described to document WAR superiority in prior clinical trials [6,23,24]. To avoid bias due to multiple comparisons, since the assessment of non-inferiority and potential superiority was assessed on overlapping and non-independent populations, a Bonferroni correction was performed, reducing the effective α threshold in the test.

#### 2.9.2. Treatment of Missing Values

Adopting a prudent approach, for the assessment of the primary and secondary objectives defined in the Clinical Investigation Plan, all missing values were imputed by propagating the last observation carried forward (LOCF). The same technique was applied to patients who dropped out due to wound worsening or complete wound healing.

#### 2.9.3. Study Populations

Considering the possibility of patient withdrawal, the occurrence of AEs and protocol deviations, the following populations were predefined at the Clinical Investigation Plan:

1. Safety study population (Safety): All randomized patients who received at least one application of the study product.
2. Intention-to-treat study population (ITT): All patients randomized and treated at least once from whom at least one post-treatment evaluation (digital picture) of the primary efficacy endpoint was obtained, whether or not they met the inclusion/exclusion criteria.
3. Per protocol study population (PP): Patients who have fulfilled the inclusion/exclusion requirements and the rest of the activities foreseen according to the protocol:
  (1) The study/control product had been applied in >80% of the visits foreseen in the protocol (minimum 20 applications) or had achieved complete healing.
  (2) Would have undergone one loss in the scheduled evaluation visits at most.

##### Baseline Values: Descriptive Analysis

A statistical description was made for all variables involved in the study, including measures of central tendency (mode, median, and mean), as well as dispersion (range and standard deviation). Baseline comparability of the two groups was verified using adapted tests (Student’s *t*-test, nonparametric Mann–Whitney test, and chi-square test), dependent on the distribution and the nature of the variables. Since the Shapiro–Wilk test concluded that quantitative variables were not consistent with normality, the nonparametric Wilcoxon-Mann–Whitney test was used to assess the homogeneity of the baseline variables with respect to the group (control and intervention). In addition, the homogeneity of the variances in the various groups to be compared was assessed using the Levene test (Brown–Forsythe in highly skewed distributions), due to the greater robustness conferred using medians versus means. Regardless of the result of the above tests, in order to facilitate data evaluation, both means and medians were shown for all variables in the study.

Qualitative and ordinal variables were described using frequency tables (absolute and relative) and the homogeneity of these baseline variables with respect to the group (control and intervention) was investigated, determining those subpopulations for which there were significant differences in the distributions using the chi-square method (χ²).

##### Efficacy Outcomes Analysis

All hypothesis tests set the α significance level at 0.05, except for the bilateral hypothesis of non-inferiority (0.025). Determination of non-inferiority and potential superiority of the study treatment was based on 95% confidence interval (95% CI) analysis on relative WAR. Considering the large deviation of wound regression variable distributions from normal and the difficulties in normalizing these distributions, only nonparametric Mann–Whitney tests were used for between-group comparisons of quantitative variables. The comparison of qualitative variables between groups was carried out using the χ² test or the Fisher’s exact test in those cases where the contingency tables presented frequencies of less than 5. 

For subgroup comparisons (assessment of the difference in WAR according to the type of ulcer), the two-way ANOVA test was used. Similarly, a repeated measures ANOVA test was used to compare the evolution of pain between treatments. Moreover, variables involving time evolution were analyzed using a Kaplan–Meier approach and followed by Log-Rank curve comparison test. Complementarily, the Cox regression proportional hazards model was applied to assess variation in WAR, considering time as a covariate. 

Scale variables are presented by their mean ± SD, median, and range. Median differences are given with 95% CIs according to the method proposed by Bonett and Price [25]. Ordinal and nominal variables are presented by the number of patients involved and percentage.

## 3. Results

### 3.1. Baseline Characteristics

**Figure 1:**
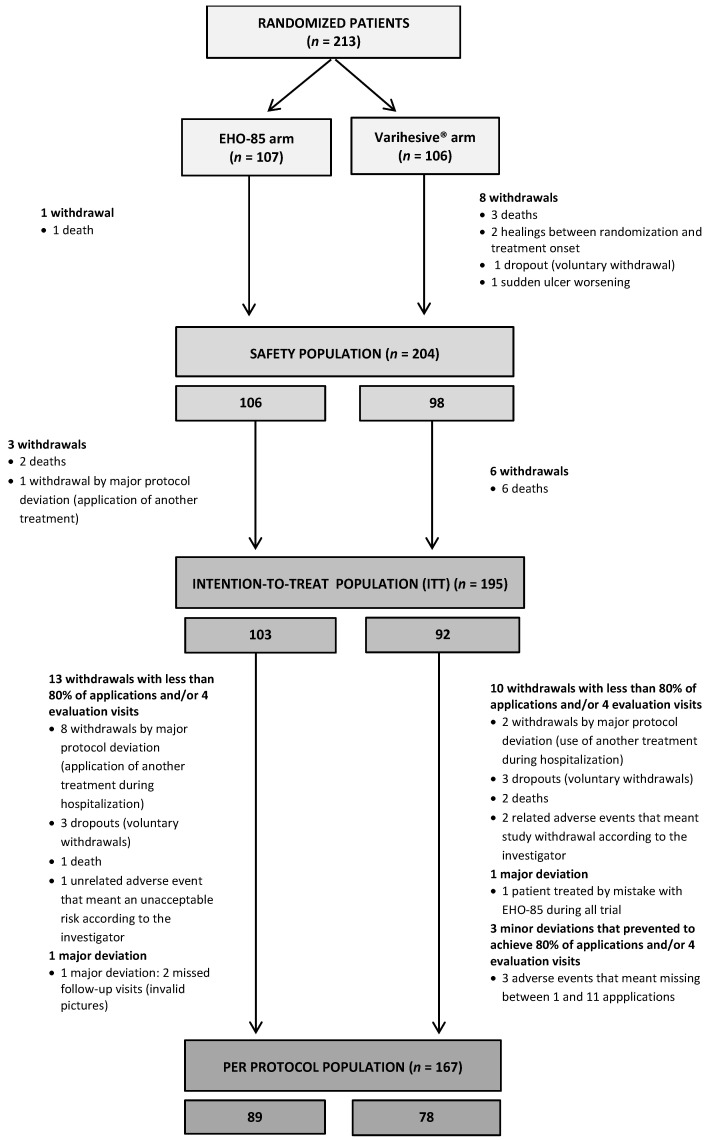
Patient flow diagram. All socio-demographic data, ulcer characteristics, and prior local treatments were well balanced between the two groups at baseline (Table 1 and Table 2), without any significant differences between their mean or median values. The same applied to PU, VLU, and DFU when their baseline data were analyzed separately.

From November 2018 to June 2019, 213 patients from 23 health centers were included in the study and randomized to receive either the EHO-85 gel (*n* = 107) or the positive control (*n* = 106). Of these, 18 patients could not be included in the ITT analysis: 4 in the EHO-85 group and 14 in the control group, mainly due to the death of patients (67%) (Figure 1). Therefore, 195 patients (92%) who had received at least one application of either treatment under investigation, and from whom at least one post-treatment digital picture had been obtained, made up the ITT analysis population. In total, 28 patients (14 in each group) were excluded from the PP analysis population, which gathered the 167 patients in whom the study/comparator treatment had been applied at least 20 times (or in whom complete healing was achieved) and who would have missed one scheduled evaluation visit at most. Following best practice, the non-inferiority analysis was conducted in the PP population. Conversely, the efficacy analyses oriented towards superiority were performed in the ITT populations. In any case, no relevant differences between the PP and ITT analyses were found for any endpoint.

PUs were the most common (60.1%), followed by VLUs (34.3%) and DFUs (5.6%). Most ulcers were unique (63.1%) and 32.4% were recurrent. Ulcers had been present for 7.7 months on average (median: 4 months; range: 1–36 months) and had an area equal or less than 10 cm^2^ (72.8%). Granulation tissue covered, on average, 75.2 ± 38.4% and the majority of ulcers presented medium (35.9%) or low (47.2%) exudate levels.

In line with clinical practice guidelines, the majority of subjects (80.0%) were being treated with moist wound care dressings and 56.9% had required any kind of debridement within the 30 days prior to trial treatment onset, mostly enzymatic and/or autolytic. Likewise, 34.4% required treatment to manage wound infections. Remarkably, most of the VLU were being treated with compression therapy (95.8%). In parallel, a large majority of patients presenting with PU used pressure-relieving mattresses (93.8%) and followed repositioning protocols (99.1%) prior to the start of the trial treatments.

### 3.2. Wound Area Reduction (WAR)

**Figure 2:**
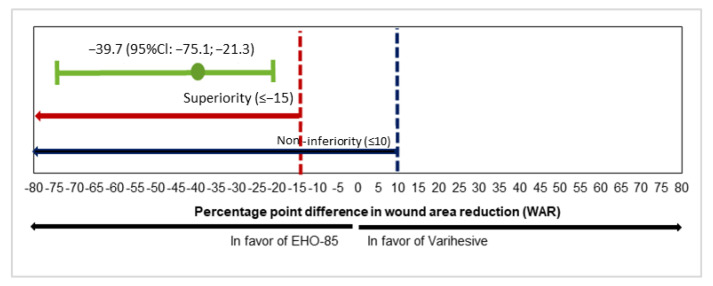
Difference between medians of relative reduction of ulcer areas with EHO-85 and the positive control at the end of the study. Non-inferiority threshold (blue marked): 10% in favor of VariHesive^®^; Superiority threshold (red marked): −15% in favor of EHO-85. Intention-to-treat analysis. The chart displays the 95%CI of the difference between medians.

**Figure 3:**
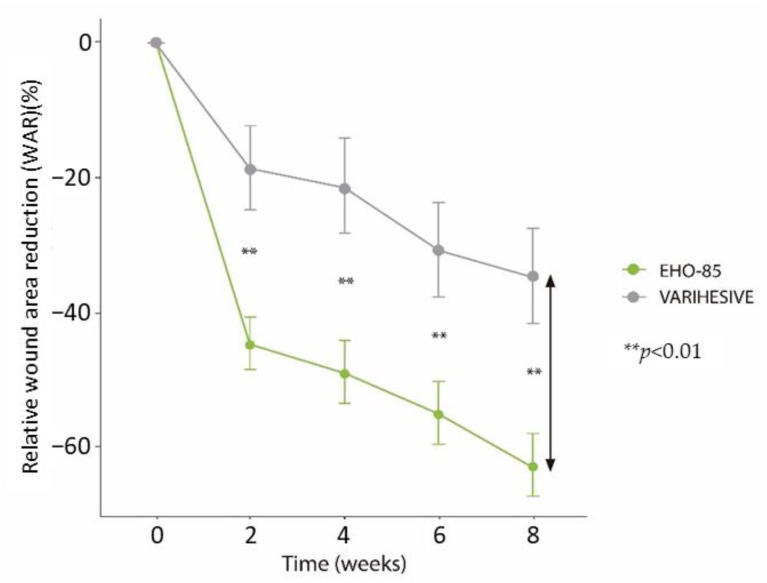
Evolution of the reduction in percentage of the ulcer area in both treatment groups during the 8 weeks of follow-up. Intention-to-treat analysis. Results are expressed as mean ± SD. ** *p* < 0.01. The arrow shows the primary endpoint of the study.

The primary objective, the median relative WAR, decreased by −79.4% in the EHO-85 group and by −34.6% in the control group (difference: −39.72%; 95% CI for median difference: −75.1 to −21.3%; *p* < 0.001), as presented in Figure 2. The lower limit of this confidence interval was well above the non-inferiority (10%) and superiority (−15%) thresholds, thereby demonstrating the superiority of the EHO-85 gel compared with the positive control. The reduction of the ulcer area with EHO-85 was statistically significant throughout the study (*p* < 0.01 at all time points) (Figure 3).

**Figure 4:**
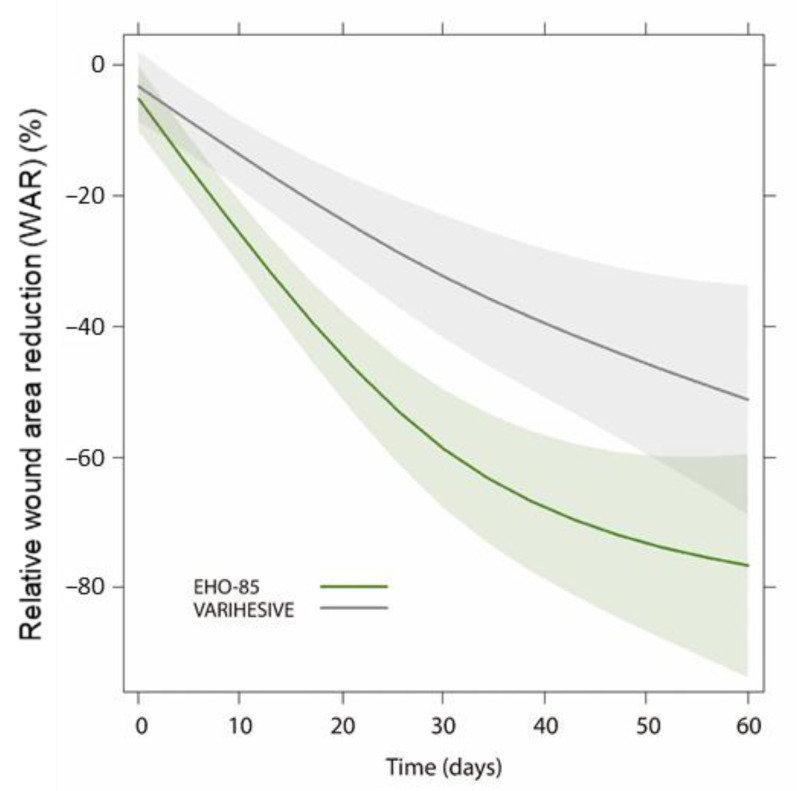
Smoothed curves to show the reduction in ulcer area with EHO-85 and comparator.

Similarly, results obtained from the multivariate WAR model, designed to support the conclusions of the main endpoint, confirmed that the EHO-85 gel promotes and accelerates the healing process of skin ulcers and that it does so in a significantly superior manner to the comparator. The multivariate model allows us to conclude that the application of the two treatments produces a significant reduction in the ulcer area (*p* < 0.0001), although the effect of the EHO-85 gel on wound healing is superior from the first weeks evaluated and until the end of the 8-week follow-up period (*p* = 0.0086) (Figure 4).

### 3.3. Secondary Endpoints

Table 3 contains results of complementary endpoints to document whether the EHO-85 gel promotes and/or accelerates the healing process of skin ulcers.

**Figure 5:**
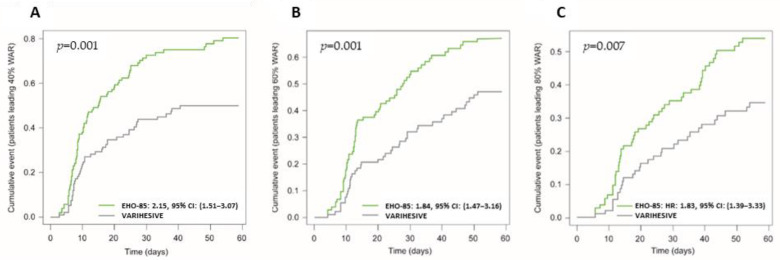
Kaplan–Meier curves. Cumulated incidence of patients with ulcer healing ≥40% (**A**), ≥60% (**B**) and ≥80% (**C**). Intention-to-treat analysis. (HR) Hazard Ratio. (CI) Confidence Interval.

Consistent with the primary endpoint results, highly statistically significant differences were observed in favor of the EHO-85 gel for absolute WAR (*p* = 0.002) and daily healing rate (*p* = 0.002). Moreover, despite a clinically important difference in the number of patients reaching complete ulcer healing (34 patients [EHO-85] vs. 22 patients [positive control]), the difference was not statistically significant. However, WAR of more than 40% from baseline value (WAR ≥ 40%) was significantly higher in the EHO-85 group (78 vs. 45 patients; *p* < 0.001). Accordingly, more stringent criteria (WAR ≥ 60% and WAR ≥ 80%) implemented in a post hoc analysis confirmed the superiority of the investigational treatment over the positive control (64 vs. 41 patients, *p* = 0.01 and 51 vs. 30 patients, *p* = 0.02, respectively). In this regard, Kaplan–Meier analysis concluded that treatment with the EHO-85 gel was associated with a greater likelihood of achieving WAR ≥ 40%, being more than twice as likely to reach these levels of wound closure using the investigational treatment compared to the positive control (Figure 5). Similar ratios were documented for WAR ≥ 60% and WAR ≥ 80% (Hazard Ratios 1.84 and 1.83, respectively). Contrarily, no relevant differences were found in the median number of days to reach WAR ≥ 40% or 100%.

### 3.4. Subgroup Analysis: Treatment Efficacy According to Ulcer Etiology

Before starting the efficacy evaluation of the investigational treatment on each type of ulcer separately, it was assessed whether relevant differences in WAR existed among the different etiologies evaluated (PU, VLU and DFU) when treated with the EHO-85 gel. No difference on the wound healing ability of the new treatment was found according to ulcer type (p_ANOVA_ = 0.293). The EHO-85 gel is, therefore, equally effective for all types of ulcers.

**Figure 6:**
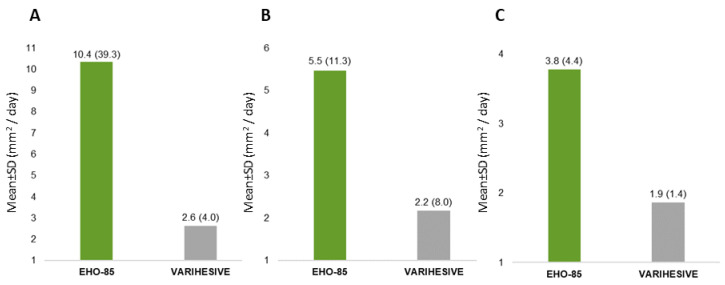
Mean daily reduction of ulcer area by ulcer type. Intention-to-treat analysis. Results are expressed as mean ± SD. (**A**) pressure ulcer; (**B**) venous leg ulcer and (**C**) diabetic foot ulcer.

Differences in WAR between the EHO-85 gel and comparator appear clinically important irrespective of ulcer etiology (Table 4; Figure 6). In the future, separate randomized clinical trials should be conducted for each type of ulcer.

However, statistical significance on relative WAR was documented only for PU. As it is usually the case for subgroup analysis, VLU and DFU sample sizes were insufficient (72 and 10 ulcers, respectively) given the dispersion of the results.

### 3.5. Ulcer Pain or Discomfort Relief

**Figure 7:**
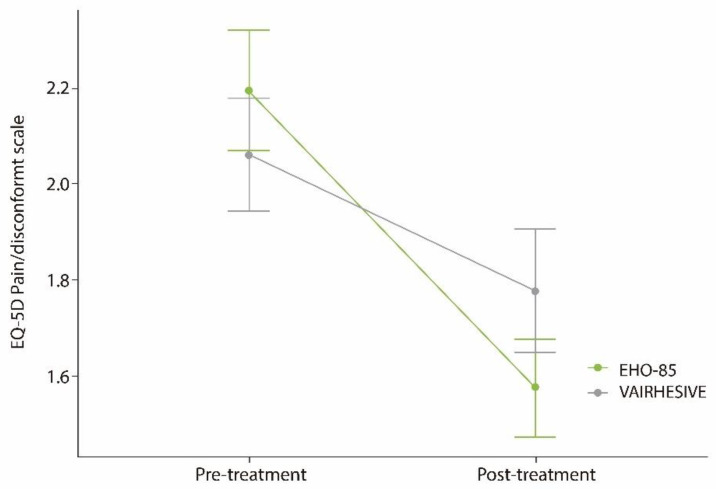
Ulcer pain before and after treatment as assessed by the EQ-5D-3L questionnaire. Results obtained from the responses obtained from patients who were able to complete the question referring to perceived pain/discomfort in relation to the ulcer (*n* = 105). Scale from 1 (no pain/discomfort) to 5 (extreme pain/discomfort). Responses collected at initial (pre-treatment) and final (post-treatment) visits.

A significant reduction in pain and discomfort in the ulcer was associated with both treatments (*p* < 0.001 in the EHO-85 group and *p* = 0.022 in the control group). The interaction effect between time and treatment showed a slightly higher superior effect on pain reduction for treatment with the EHO-85 gel (Figure 7), probably due to its increased healing capacity. Nonetheless, this greater pain and discomfort reduction did not reach statistical significance (ANOVA test).

### 3.6. Granulation Tissue Formation

**Figure 8:**
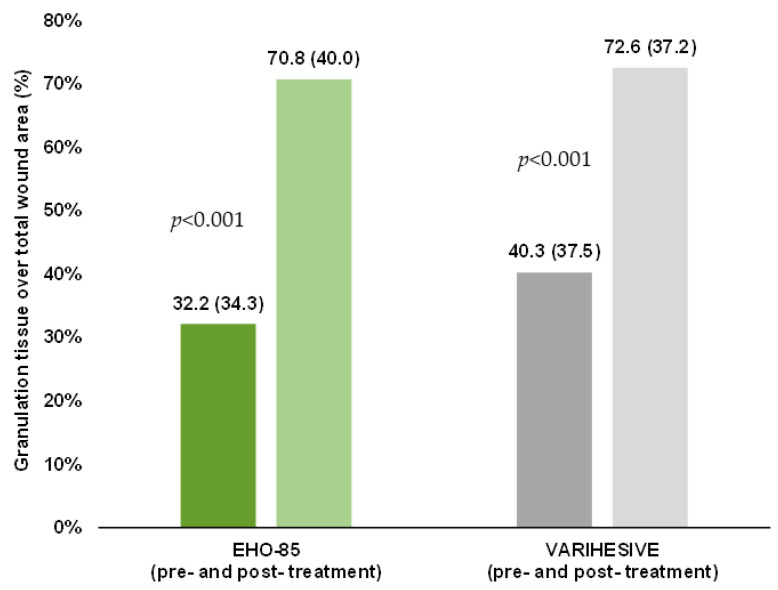
Percentage of granulation tissue over the total ulcer area before and after treatment (intra-group differences). The sample comprises only patients with ulcers with a percentage of granulation tissue less than 100% at the baseline visit (EHO-85, *n* = 44; VariHesive^®^, *n* = 31). Intention-to-treat analysis. Results are expressed as mean ± SD.

Both treatments significantly promoted the formation of granulation tissue (*p* < 0.001), in accordance with what would be expected for an amorphous hydrogel. Granulation tissue formation was slightly higher in the EHO-85 group, without reaching statistical significance (Figure 8).

### 3.7. Cost Effectiveness: Amount of Product Needed to Fulfill the 8 Weeks of Treatment

This analysis was foreseen to be made as per-protocol, since this group of patients showed greater adherence to the treatment guidelines set out in the Clinical Investigation Plan and achieved at least 80% of the total expected product applications throughout the 8-week follow-up period. The average number of 15 g packs used per patient during the study was 2.69 ± 2.33 in the EHO-85 gel group and 20.15 ± 6.39 in the positive control group (*p* < 0.001).

### 3.8. Safety

#### 3.8.1. Total Adverse Events

The total number AEs recorded by the investigators among the safety population (203 patients treated at least once, regardless of whether a post-treatment efficacy assessment could be made) amounted to 68, with no relevant differences documented between the two treatment groups: 32 in the EHO-85 group vs. 36 in the comparator group. No probable or possible causal relationship was found between the 23 severe AEs and the study treatments.

#### 3.8.2. Local Adverse Events

None of the local AEs were considered serious. The median number of days from the start of treatment to occurrence of the local AE was 20.9 days among patients treated with EHO-85, compared to 21.8 days in the control group. Twenty-seven local AEs were noted (Table 5), with no relevant differences between the two treatment groups: 11 in the EHO-85 group vs. 16 in the control group.

**Figure 9:**
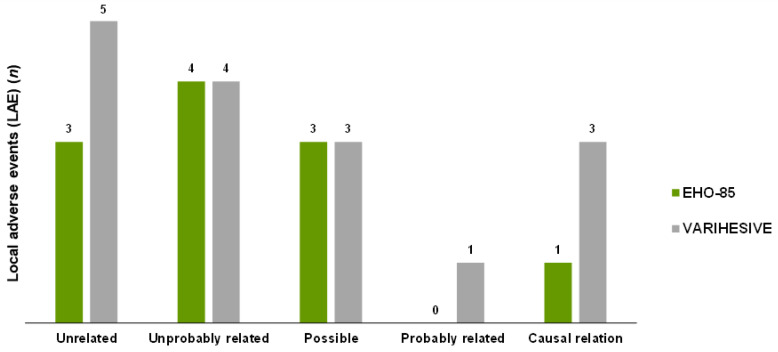
Relationship between local adverse events and treatments.

Wound infection was the most frequently reported local AE (15 out of 27 local AEs in total), with a much higher incidence in the comparator treatment group (10 vs. 5). Only 4 of the 27 local AEs documented were considered by the investigators to be “causally treatment related” or “most probably treatment related” (one in the test group and four in the control group) (Figure 9).

For two patients in the test group and three patients in the control group, the local AE was the reason that justified discontinuation of the treatment application before week 8. Similarly, study treatments were temporally discontinued in four patients in the control group due to local AEs (none in the EHO-85 gel group).

## 4. Discussion

The new EHO-85 gel was designed to be capable of combining the moistening and barrier-function properties of any amorphous hydrogel with the novel ability to downregulate ROS and pH, thus modulating the microenvironment of the ulcer in a holistic manner. To date few studies have focused on demonstrating that modulation of the wound microenvironment—in conjunction with the best standards of care—may promote the healing process of skin ulcers [6,7]. This 8-week, multicenter, randomized, active-controlled and observer-blinded clinical trial was designed to document the non-inferiority or superiority of the novel EHO-85 gel relative to a reference hydrogel (VariHesive^®^), currently used safely in standard practice worldwide, for the treatment of skin ulcers.

To confirm the clinical relevance of potential differences between the effect of the EHO-85 gel and the comparator, strict inclusion and exclusion criteria were implemented and best practices in ulcer local management were applied identically in both groups [26,27,28]. In this regard, in compliance with the use of compression bandaging (VLU), the use of felted foam and appropriate footwear (DFU) and repositioning (PU) was very high throughout the 8-week trial (8 minor deviations for this reason in the experimental group compared to 6 in the comparator group), thus minimizing potential bias from not using these measures.

The main efficacy criterion in our study was the decrease in the wound area from baseline or relative WAR. Change in the ulcer area after 8 weeks of therapy is considered a surrogate marker to predict healing of chronic wounds [29,30] and it has been used increasingly in clinical trials of skin ulcer therapy [6,23,31]. Despite the fact that the study was open, this criterion was evaluated centrally and blindly by an independent researcher and the statistical analysis was conducted by an independent entity. Results for the primary endpoint documented the superiority of the EHO-85 gel vs. positive control, with clinically significant and continued improvements in the relative WAR from the first weeks of application. The difference in median WAR between hydrogels was almost 40% in favor of EHO-85, which represents a 2-fold increase in the WAR rate. It is far better than the 15% proposed as being clinically relevant in the literature [6,23]. Other secondary endpoints designed to document whether EHO-85 accelerates the healing process of skin ulcers—such as absolute WAR and daily healing rate—reinforced that hypothesis. Moreover, the effect of applying the gel under study on WAR did not vary depending on ulcer etiology (PU, VLU and DFU) (p_ANOVA_ = 0.293). Another secondary outcome was the number of patients reaching complete ulcer healing (34 patients treated with *O. europaea* gel vs. 22 patients treated with the positive control). Although the difference might be clinically important, it was not statistically significant. With regard to this point, the main limitation of the study was the follow-up duration. According to previous studies [6,23], 8 weeks might not be a long enough period to detect statistically significant differences in the percentage of patients with complete (100%) healing. As we observed significant differences in the number of patients with WAR ≥ 80%, we would probably have found statistically significant differences with a longer follow-up of 10 or 12 weeks. However, as already stated, change in the ulcer area after 8 weeks of therapy is considered a surrogate marker to predict healing of chronic wounds [29,30]. Some authors have suggested even shorter periods of 4–6 weeks [30,32]. In this regard, WAR in the EHO-85 gel group was 56.06% (±42.03) after 4 weeks of therapy. Accordingly, the secondary endpoint assessing the number of patients achieving WAR ≥ 40% after 8 weeks of treatment was significantly higher in the EHO-85 group (78 vs. 45 patients; *p* < 0.001). Such reduction in wound area within the first weeks of treatment is highly predictive of a fully healed ulcer within 20–24 weeks [29,32,33,34,35]. Accordingly, the percentage of patients achieving WAR ≥ 40% after 56 days has been used as an endpoint in recent RCTs on wound healing [6,23].

Likewise, a set of secondary outcomes was designed to evaluate other potential benefits consistent with the design of the novel EHO-85 gel: As expected from an amorphous hydrogel, it significantly promoted granulation tissue formation and significantly diminished wound and peri-wound pain. It did slightly better than the positive control. However, between-group differences did not reach statistical significance.

Furthermore, the new EHO-85 gel incorporates a very well-tolerated preservative in its formulation at a low concentration, in addition to undergoing a sterilization process. This enables a very significant reduction in the number of product units to be used per patient compared with single-dose amorphous hydrogels (−85% 15 g units), rendering the EHO-85 gel into a cost-efficient treatment which eliminates the widespread waste of hydrogels. Interestingly, the addition of the preservative and other novel compounds such as the *Olea europaea* leaf extract does not interact with the overall local tolerance and acceptability of this multifunctional amorphous hydrogel, as no between-group differences were documented in terms of safety.

These results may suggest that the addition of ROS and pH modulation attributes to those already inherent to amorphous hydrogels significantly increases the wound healing capacity of these dressings previously shown and documented [13], thereby converting the EHO-85 gel into a safe and cost-effective active dressing able to also cover all the requirements of an amorphous hydrogel. This study is particularly relevant because, as far as we know, hitherto there was no evidence from randomized controlled trials on hydrogels for ulcer healing. Furthermore, previous to this study, no hydrogel was proven to be superior to other hydrogels.

In conclusion, the present clinical investigation is a relevant milestone in evidence-based practice since it is the first time that a randomized clinical trial has demonstrated (i) the effectiveness of the application of an amorphous hydrogel in the acceleration of wound healing and (ii) the superiority of a specific hydrogel over another hydrogel. EHO-85 gel is more effective than usual care and can, therefore, be considered a first-in-class multifunctional amorphous hydrogel, with an excellent safety profile, that is cost efficient due to its multi-dose format.

## Figures and Tables

**Table 1:** Baseline Characteristics of Patients.

Characteristic	EHO-85 (*n* = 103) Mean ± SD or *n* (%)	VariHesive^®^ (*n* = 92) Mean ± SD or *n* (%)	*p*
**Sex**, women	70 (68.0%)	59 (64.1%)	0.68
**Age**, years	78.0 ± 13.1	79.5 ± 14.8	0.18
**BMI**, kg/m^2^	27.4 ± 6.0	28.8 ± 8.1	0.36
**Ankle/brachial index (ABI)** (VLU only)	1.0 ± 0.2	1.0 ± 0.2	0.98
**Diabetes mellitus**	41 (39.8%)	30 (32.6%)	0.37
**Current smoker**	7 (6.8%)	5 (5.4%)	0.31
**Alcohol intake**	9 (8.7%)	7 (7.6%)	0.98
**Place of patient care**			0.74
Health center	22 (21.4%)	24 (26.1%)	
Own home	52 (50.5%)	44 (47.8%)	
Nursing center	29 (28.1%)	24 (26.1%)	
**Autonomy level**			0.18
He/she can walk easily	22 (21.4%)	24 (26.1%)	
He/she has some difficulty to walking	45 (43.7%)	47 (51.1%)	
Unable to walk, bedridden	36 (34.9%)	21 (22.8%)	
**Blood test** Serum album (3.40–5.0 g/dl)	3.7 ± 0.5	3.5 ± 0.5	0.13
Creatinine clearance (80–120 mL/min)	105.1 ± 46.8	107.3 ± 57.1	0.93

Most patients were women and the mean age was almost 80 years. Most patients were overweight, non-smokers and non-alcohol drinkers, lived with their families or caregivers, and had reduced mobility (Table 1). Diabetes had been diagnosed in 71 patients (36.4%).

**Table 2:** Description of Ulcers and Prior Treatments.

Characteristic	EHO-85 (*n* = 103) Mean ± SD or *n* (%)	VariHesive^®^ (*n* = 92) Mean ± SD or *n* (%)	*p*
**Etiology**			0.81
Venous	36 (34.9%)	36 (39.1%)	
Pressure	62 (60.2%)	51 (55.4%)	
*EPUAP II*	*42 (67.7%)*	*33 (64.7%)*	
*EPUAP III*	*20 (32.3%)*	*18 (35.3%)*	
Diabetic foot	5 (4.9%)	5 (5.4%)	
*Wagner I*	*2 (40.0%)*	*3 (60.0%)*	
*Wagner II*	*3 (60.0%)*	*2 (40.0%)*	
**Total number of ulcers per patient ^a^**			0.65
1	68 (66.0%)	55 (59.8%)	
2	19 (18.5%)	19 (20.6%)	
≥3	16 (15.5%)	18 (19.6%)	
**Evolution time**, months	7.1 ± 8.5	8.2 ± 9.4	0.56
**Duration** >6 months	35 (34.0%)	34 (37.0%)	0.78
**Wound area**, cm^2^	5.4 ± 9.0	3.4 ± 4.2	0.45
**Wound area** >10 cm^2^	28 (27.2%)	25 (27.2%)	1.00
**Granulation tissue**, % over total ulcer	71.0 ± 40.4	79.9 (35.6)	0.14
**Exudate**			0.82
None	14 (13.6%)	9 (9.8%)	
Low	47 (45.6%)	45 (48.9%)	
Intermediate	36 (35.0%)	34 (37.0%)	
High	6 (5.8%)	4 (4.3%)	
**Recurrent ulcer**	35 (34.0%)	31 (33.7%)	1.00
**Previous hospitalizations due to the treated ulcer**	9 (8.7%)	3 (3.3%)	0.20
**Ulcer pain intensity ^b^**			0.77
No pain nor discomfort	16 (28.6%)	14 (28.6%)	
Slight pain or discomfort	17 (30.4%)	19 (38.8%)	
Moderate pain or discomfort	19 (33.9%)	15 (30.6%)	
Intense pain or discomfort	4 (7.1%)	1 (2.0%)	
Extreme pain or discomfort	0 (0.0%)	0 (0.0%)	
**Cures during the previous month**			0.75
Cure in dry environment	16 (15.5%)	16 (17.4%)	
Cure in moist environment	82 (79.6%)	74 (80.4%)	
Both	2 (1.9%)	0 (0.0%)	
Unknown	3 (2.9%)	2 (2.2%)	
**Debridement in the last month**	45 (43.7%)	39 (42.4%)	0.97
**Sharp/surgical debridement** 24–72 h before visit 1	9 (8.7%)	16 (17.4%)	0.11

^a^ According to the planning of clinical investigation, only one ulcer per patient was treated. ^b^ Only patients able to complete the questionnaire.

**Table 3:** Secondary Wound Healing Outcomes. Intention-to-Treat Analysis.

		EHO-85 Gel (*n* = 103)	VariHesive^®^ (*n* = 92)	*p*
Absolute wound area reduction (mm^2^)	Mean ± SD	251.92 (553.61)	108.18 (247.26)	0.002
	Median (range)	115.80 (−451.0; 4187.7)	51.15 (−797.0; 1162.6)	
Daily healing (mm^2^/day)	Mean ± SD	8.33 (31.19)	2.40 (5.78)	0.002
	Median (range)	2.77 (−34.5; 298.9)	1.18 (−24.5; 20.8)	
N° of patients with complete healing (100%)	N° of patients (%)	34 (33.01%)	22 (23.91%)	0.205
N° average days until complete healing	Mean ± SD	28.68 (17.13)	28.64 (12.23)	0.794
	Median (range)	23.00 (−100.0; 111.0)	29.00 (7.0; 49.9)	
N° of patients with healing ≥40%	N° of patients (%)	78 (75.73%)	45 (48.90%)	<0.001
N° average days until healing ≥40%	Mean ± SD	14.58 (11.51)	15.31 (10.49)	0.508
	Median (range)	10.12 (2.8; 54.1)	10.43 (2.8; 40.7)	

**Table 4:** Wound Area Reduction (WAR) (%) by Ulcer Etiology.

		**EHO-85 Gel (*n* = 62)**	**VariHesive^®^ (*n* = 51)**	***p***
Pressure ulcer	Mean ± SD	−63.92 (39.95)	−37.33 (59.26)	0.007
	Median [range]	−79.05 [−100.0; 79.5]	−36.82 [−100.0; 200.00]	
		**EHO-85 gel (*n* = 36)**	**VariHesive^®^ (*n* = 36)**	***p***
Venous ulcer	Mean ± SD	−56.98 (51.22)	−28.45 (80.97)	0.196
	Median [range]	−70.39 [−100.0; 111.0]	−58.71 [−100.0; 200.00]	
		**EHO-85 gel (*n* = 5)**	**VariHesive^®^ (*n* = 5)**	***p***
Diabetic foot ulcer	Mean ± SD	−88.49 (25.75)	−51.50 (39.85)	0.058
	Median [range]	−100.00 [−100.0; −42.4]	−36.02 [−100.0; 14.57]	

**Table 5:** Local Adverse Events by Treatment.

	Total (*n* = 27)	EHO-85 Gel (*n* = 11)	VariHesive^®^ (*n* = 16)
Wound infection	15	5	10
Satelite lesions	3	2	1
Ulcer pain	2	1	1
Perilesional erythema	2	1	1
Hypergranulation	2	1	1
Wound impairment	1	0	1
Tissue bleeding	1	1	0
Perilesional maceration	1	0	1

## Data Availability

The data that support the findings of this study are available from the corresponding author upon reasonable request.

## Supplementary Materials

The following supporting information can be downloaded at: https://www.mdpi.com/article/10.3390/jcm11051260/s1, Table S1: Specific exclusion criteria for each type of ulcer; Table S2: List of collaborating nurses (sub-investigators) by centers.
